# Editorial: Factors that impact the survival of non-small cell lung cancer

**DOI:** 10.3389/fonc.2023.1148340

**Published:** 2023-02-09

**Authors:** Fiona Hegi-Johnson, Kristin A. Higgins

**Affiliations:** ^1^ Department of Radiation Oncology, Peter MacCallum Cancer Centre, Melbourne, VIC, Australia; ^2^ Sir Peter MacCallum Department of Oncology, The University of Melbourne, VIC, Australia; ^3^ Department of Radiation Oncology, Winship Cancer Institute, Emory University, Atlanta, GA, United States

**Keywords:** non-small cell lung cancer (NSCLC), surgery, radiotherapy, immunotherapy, epidemiology

There has never been a more exciting time to be a thoracic oncologist.

The last decades of biological discovery have led to meaningful gains in survival through the development of EGFR tyrosine kinase inhibitors (TKIs) and immunotherapy, and as this collection demonstrates, an increasing understanding of the factors that influence survival.

This collection, of the most highly cited and popular articles published within Frontiers in Oncology in Non-Small Cell Lung cancer over the last 5 years, illustrates the great depth and breadth of this field and focuses in particular on the biology and treatment outcomes of lung cancer patients with adenocarcinomas.

As Baak et al. show, NSCLC patients are diverse, and it is increasingly clear that the paradigms to predict outcomes which are based largely upon stage, histology and treatment can no longer effectively predict survival. Their intriguing finding that biological factors, such as clonality, proliferation and intra-tumour heterogeneity may differentiate between subpopulations of patients with different survival is to some extent justified by the other articles in this collection.

Despite the startling gains in survival seen in recent immunotherapy trials in both early stage NSCLC (1, 2) and metastatic NSCLC (3) the biology of the immune response remains poorly defined, and the intricacies of this response, and the mechanisms of resistance, which develop in the majority of patients treated with immunotherapy will require a sustained commitment to overcome. To this end, it is heartening to see so much emerging preclinical data exploring the mechanisms underpinning immunotherapy response and resistance. The predictive power of novel tumour biomarker signatures are explored by Yang et al. who demonstrate that RNA sequencing of long-coding RNA may help to predict survival in adenocarcinoma patients.

Our understanding of oncogenic pathways in lung adenocarcinoma also continues to deepen, and the evaluation of CDCA4, a gene that encodes transcription factors involved in cell proliferation and DNA synthesis in a small surgical cohort of patients provides yet another pathway to be interrogated in larger datasets and preclinical models (Tan et al.). A further two papers in this collection interrogate large patient databases to assess whether genomic signatures associated with macrophage switching(Chen et al.) and TGF-beta expression in radiotherapy patients are associated with overall survival (Zhang et al.). Using genomic analysis from a database and CRISPR silencing of acetyl-CoA acyltransferase (ACAA1) Feng and Shen also investigated causes of immunotherapy response and resistance in KRAS driven NSCLC demonstrating and appears to drive the development of an immunosuppressive tumour microenvironment dominated by CD4+. T-Helper(TH)1, TH2 and TREG cells.

Imaging biomarkers continue to evolve, and offer the promise of non-invasive, multiple time-point assessments of tumour biology. Although, as pointed out by O’Connor et al. (4) CT-based radiomics still requires refinement and the use of large scale training datasets before a validated methodology is established it is encouraging to see work that employs large cohorts of patients with uniform biology to develop tools that are specific to the East Asian adenocarcinoma population (Wang et al.). Similarly, Jiang et al. review the correlations between EGFR mutation status and 18-F-FDG avidity, which also has useful real world applications for disease monitoring in patients being treated with tyrosine kinase inhibitors (TKIs).

The collection also captures the truly multidisciplinary nature of NSCLC management, and deals not only with improving survival but also with treatment and prognostication in patients with pleural effusions(Li et al., Qiao et al.) and with the ability of non-medication based approaches to improve analgesia and quality of life (Tang et al.).

In early stage disease, useful data is explored that looks at disease and treatment characteristics influencing survival in both surgical (Huang et al.) and radiotherapy(Yang et al., Li et al.) patients with both adenocarcinoma and squamous histologies. Given the paucity of clinical trial data that is specific to the histological subtypes of adenocarcinoma and squamous cell carcinomas, these retrospective analyses provide some useful guidance to support changes that are occurring in clinical practice to de-intensify local treatment approaches, such as the movement towards segmentectomy rather than lobectomy.

The efficient and rapid evaluation of emerging treatment strategies in clinical trials is handled in a different way by a paper exploring the value of early-stage endpoints as surrogate markers for later endpoints of durable response such as long-term progression free and overall survival (Shameer et al.). As the authors point out the sue of early end-point surrogates, permitting the assessment of survival outcomes at 4 or 6 months holds great promise to reduce the cost of clinical trials but needs significant future work in this area before these strategies can be validated for wider clinical use.

Finally, to underscore the importance of prevention, an increasing body of data underscores the importance of smoking cessation on both cancer causation and mortality after diagnosis. As Koshiaris (5) and Jha (6) have pointed out smoking accounts continues to account for an enormous proportion of cancer deaths, with an increasing burden falling on low- and middle-income countries. However, there is relatively little data on the interplay between smoking and response to therapies. Given the high levels of both EGFR expression and the high incidence of smoking in the East Asian population it is encouraging to see the exploration of this relationship, although the finding that smoking is a less powerful driver of response to TKI’s than gender raises further intriguing biological questions to be explored in future work (Xiao et al.).

In summary, the future is bright for non-small lung cancer, as the thoracic oncology community continues to work together to advance the science for our patients.

## Author contributions

FH-J and KH have drafted and reviewed this manuscript prior to submission. All authors contributed to the article and approved the submitted version
